# Ingrowing Liver as Atypical Recurrent Diaphragmatic Hernia Presentation—Diagnostic and Treatment Difficulties: A Case Report

**DOI:** 10.3390/pediatric14010020

**Published:** 2022-03-11

**Authors:** Dominika Borselle, Krzysztof Międzybrodzki, Sylwester Gerus, Urszula Zaleska-Dorobisz, Agnieszka Hałoń, Leszek Szenborn, Dariusz Patkowski

**Affiliations:** 1 Department of Pediatric Surgery and Urology, Wroclaw Medical University, 50-556 Wrocław, Poland; sylwester.gerus@umw.edu.pl (S.G.); dariusz.patkowski@umw.edu.pl (D.P.); 2 Department of General and Pediatric Radiology, Wroclaw Medical University, 50-369 Wrocław, Poland; krzysztof.miedzybrodzki@umw.edu.pl (K.M.); urszula.zaleska-dorobisz@umw.edu.pl (U.Z.-D.); 3 Department of Pathomorphology, Wroclaw Medical University, 50-556 Wrocław, Poland; agnieszka.halon@umw.edu.pl; 4 Department of Pediatric Infectious Diseases, Wroclaw Medical University, 50-368 Wrocław, Poland; leszek.szenborn@umw.edu.pl

**Keywords:** ingrowing liver, CDH recurrence, thoracoscopy, minimally invasive surgery

## Abstract

(1) Introduction: Recurrent diaphragmatic hernia is a relevant diagnostic and treatment dilemma. We have presented a patient with ingrowing liver as an atypical diaphragmatic hernia recurrence and discussed major aspects of diagnostic methods and the selection of an appropriate operative treatment. (2) Case description: We discuss a case of a patient with right-sided recurrent CDH (Congenital Diaphragmatic Hernia) who had primary thoracoscopic repair in newborn period. During infancy and early childhood, the patient presented recurrent upper and lower respiratory tract infections and bronchial hyperreactivity. The clinical picture was initially unclear. A CT scan was inconclusive to diagnose a recurrence. The patient was scheduled to have a re-thoracoscopy. A part of the liver was herniated into the pleural cavity. This fragment of ‘ingrowing’ liver was removed, and the diaphragmatic secondary defect was repaired. (3) Conclusions: This case proved that thoracoscopy can be a preferred technique in the diagnosis and treatment of CDH recurrence.

## 1. Introduction

Recurrent diaphragmatic hernia is a relevant diagnostic and treatment dilemma that has been reported in the literature. The recurrence rate in the series available is described in a wide range of cases, and it seems to be higher for endoscopic approach—7.9% [1,2,3]. 

The recurrence of a diaphragmatic hernia is usually apparent in chest radiographs. Other patients require a US or CT/MRI (Ultrasonography or Computed Tomography/Magnetic Resonance Imaging) scan to confirm a diagnosis [4]. The reasons for diagnostic and treatment challenges of CDH recurrence include the frequency rate, the different clinical and radiological pictures and the diversity of surgical techniques. 

The aim of this study is to present a case of an atypical recurrent CDH in a 4.5-year-old patient and to discuss the difficulties in the diagnostic process, the selection of an appropriate operative treatment and the atypical mechanism of recurrence manifested as an ‘ingrowing liver’.

## 2. Case Presentation

The 4.5-year-old girl was operated on within the first day of her life. A right-side thoracoscopy was performed, during which bowels loops and the right liver lobe were relocated into the peritoneal cavity. The medium-sized 3 cm × 3 cm (type B) diaphragm defect was primarily closed with non-absorbable interrupted sutures. The right lung appeared to be hypoplastic during the procedure.

During infancy and early childhood, the patient presented recurrent upper and lower respiratory tract infections and bronchial hyperreactivity. In the beginning, the symptoms included coughing, wheezing, shortness of breath and abnormalities in the physical examination, which was completely withdrawn after drug treatment. The symptoms intensified seasonally, and they became more frequent and resistant to the symptomatic treatment and antibiotics. The patient’s psychomotoric development and weight were adequate. Conventional X-rays did not reveal anything specific. The patient was diagnosed with bronchial asthma, allergy and mild stenosis of the left main bronchus, revealed in bronchoscopy. At the age of four, the patient went down with lobar pneumonia (Table 1). 

## 3. Diagnostics and Therapeutic Intervention

**Figure 1:**
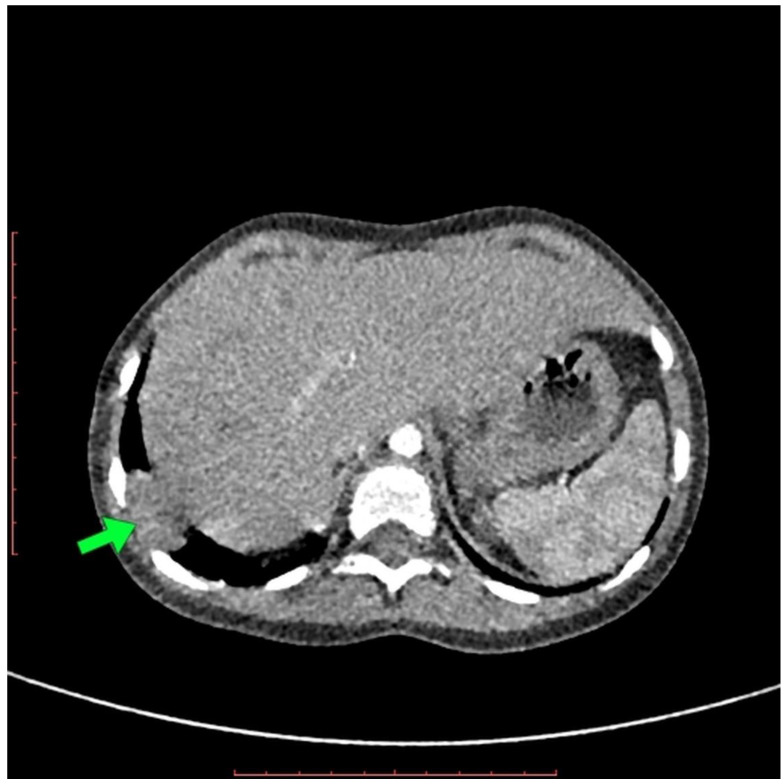
The CT scan in axial projection shows a small, rounded structure in the right dome of the diaphragm with contrast enhancement similar to liver tissue (arrow).

The X-ray revealed areas of lung inflammation typical for pneumonia. On ultrasound examination during the control visit, a structure 17–18 mm of right, supradiaphragmatic localization was described, with suggestions of recurrent hernia, postoperative residue, or lesion with other etiology. Due to an inconclusive result, a CT scan of the thorax was performed. The CT examination was performed using a 128-slices CT scanner and a low-dose scanning technique under general anesthesia. The CT examination revealed a small, rounded structure with density and contrast enhancement similar to liver tissue in the right dome of diaphragm. The CT examination did not reveal the presence of any accompanying pathological opacities in the surrounding pulmonary parenchyma or the presence of any fluid in the right pleural cavity (Figure 1). The examination suggested segmental diaphragm relaxation (Figure 1).

There was no complication in postoperative period. The hospitalization length was four days. According to a minor size of diaphragmatic defect and herniation part of liver, the prognoses of respiratory symptoms have been good, and the previous symptoms might rather have been caused by lung hypoplasia, diaphragmatic impairment and other reasons.

Due to the divergent results of the ultrasound and CT, the patient was scheduled for diagnostic re-thoracoscopy. 

**Figure 2:**
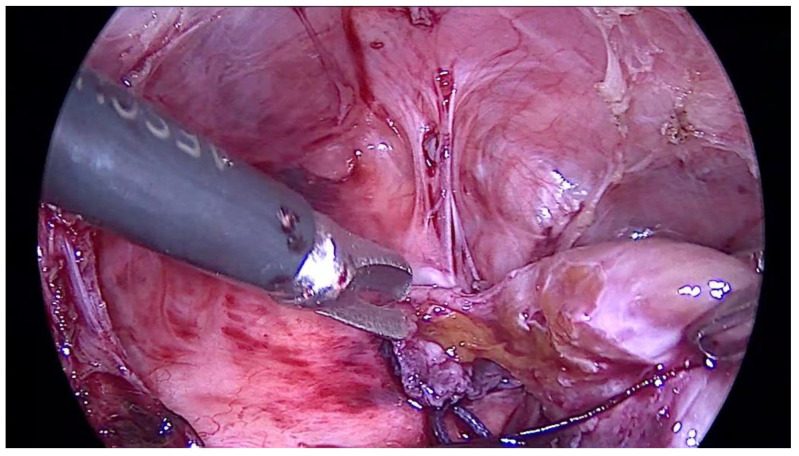
The picture shows a spherical structure with narrow neck penetrating into the diaphragm and macroscopically similar to liver tissue.

**Figure 3:**
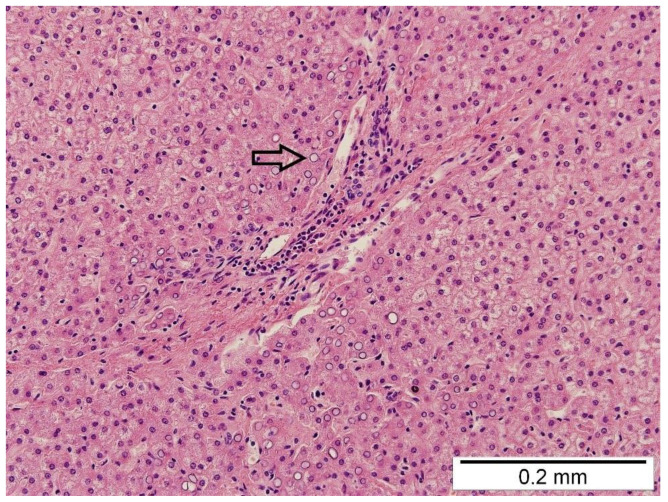
The histopathological examination shows the hepatocytes arranged in one-cell-layer-thick plates separated by sinusoids. No inflammatory cells, no fibrosis, and no steatosis were observed. A few glycogenated nuclei were observed (arrow).

In the right pleural cavity, the surgeon cut many adhesions and identified a spherical, 2 cm × 2 cm sized structure with a narrow neck that penetrated into the diaphragm (Figure 2). Macroscopically, the structure was similar to liver tissue. This neck structure was ligated and the ingrown liver tissue was removed, which revealed a small, 1–1.5 cm sized, rounded defect in the diaphragm that was closed with three non-absorbable interrupted sutures. The operation time was 1 h and 40 min, and it was without blood loss. Histopathological examination confirmed the diagnosis of liver tissue with typical microarchitecture (Figure 3). 

## 4. Discussion

Diagnostic difficulties presented in this case are the main aspect to discuss. The repeated conventional chest radiographs did not reveal any pathologies, except for inflammatory changes. Only the US showed supradiaphragmatic pathology with an inconclusive suggestion; however, this method depends on the experience of the radiologist performing the examination [4]. In many cases, the CT scan might have shown discontinuity in the right posterolateral part of diaphragm, which is typical for the diaphragmatic hernia [4]. It should be noted that the CT scans of our patient did not reveal the evident discontinuity part of the diaphragm, and only a small part of the right lobe of the liver was located in the lung parenchyma. In this case, recurrent CDH was misdiagnosed in CT, and the radiologist suggested the diagnosis of segmental diaphragm relaxation. It is reported that some authors suggested that the correct differentiation between these pathologies in CT can often only be made in surgery [5]. 

In our case, the thoracoscopy made it possible to confirm the atypical recurrent CDH, to repair the defect in diaphragm at the same time and to take the tissue to histopathological examination. The thoracoscopy enabled distinguishing liver tissue from the diaphragm which was inconclusive in CT because of the similar density and contrast enhancement of both tissues. MIS techniques provided a very precise, detailed, enlarged picture of anatomical structures [6], so that a small part of the liver could be identified among many adhesions. The decision of partial liver excision was possible due to the perfect endoscopic visualization of the narrow neck of the structure. 

Another aspect to discuss is the mechanism of formation of the recurrent diaphragmatic hernia in this case, which determined the unusual clinical picture and development over time. During the first thoracoscopy, the defect was completely primarily closed by several interrupted non-absorbable sutures. The re-thoracoscopy confirmed that secondary defect was very small, and the sutures were still on site. The size of the defect was similar to a narrow neck of the structure and lower than the main part of the herniation liver. It may indicate a different mechanism of recurrence and arising the herniation. The concept of ‘ingrowing liver’ may be a possible explanation. The space between primary sutures or thin, fibrous tissue in localization of repaired defect could be a gate of recurrence. The liver has regenerative potential, so it could probably grow up and into the pleural cavity. The radiologist did not describe any pleural effusion or inflammatory-atelectatic lesions, which are typical for recurrence. This proves the small size of secondary defect. In our case, the intrathoracic part of liver was very small. Probably, if the ingrowing liver, with its proliferative potential, had enlarged in the thoracic cavity over time, it would have cause respiratory symptoms as the result of lung compression. 

An interesting, different concept of liver presence in thoracic cavity is the hepatic heterotopia, classified into four types. The literature suggested some types are associated with CDHs, such as small, accessory liver lobe connected with the main liver, an isolated ectopic liver forming a macroscopic nodule and a microscopic, ectopic liver tissue [7,8]. It has been reported that hepatic heterotopia in the diaphragm may lead to CDH recurrence [7,8]. Ectopic liver tissue consists of microscopically normal cells, but their spatial structure and metabolism may be modified by, e.g., reduced blood supply and biliary system [9]. In our case, the first thoracoscopy did not reveal any accessory liver in the thoracic cavity or connected with the main liver. The histopathological examination revealed typical, normal liver parenchyma with typical hepatic microarchitecture. Most likely, the liver ingrowth in our patient was possible through a minor space between the primary sutures. The liver, with its proliferative potential, could grow up into the pleural cavity, through the small defect in the diaphragm, leading to the recurrence. The primary sutures were intact, and the herniation liver had a limited size and a shape with a narrow neck, similar to the extent of the defect. This may explain the atypical mechanism of diaphragmatic hernia recurrence in this case. Our observation indicates that there is a possibility of liver ingrowth between suture lines, as it was in our case. However, our hypothesis has some limitations: – the histhopathological essay confirmed a kind of tissue with specific microarchitecture but not the mechanism of pathology formation. The clinical picture, intraoperative visualization of diaphragmatic defect, data from the first thoracoscopy, details from other examinations (bronchoscopy, CT scan) and literature reports may prove the unusual mechanism of diaphragmatic hernia recurrence with liver ingrowth. 

The unconventional clinical picture involves the age at diagnosis and non-specific, recurring respiratory symptoms. The respiratory symptoms can occur as a consequence of lung hypoplasia [10] and diaphragm impairment. In our case, the right lung appeared to be hypoplastic at operation. There is a higher incidence of obstructive airway disease and restrictive lung function pattern in the long-term outcome after CDH repair [10]. More recurrences appear during the first year [1] and manifest with acute gastrointestinal or respiratory symptoms or both. Moreover, these manifestations may occur in common for childhood diseases such as bronchial asthma, allergies or GERD. In our case, the unconventional clinical picture could be the result of pulmonary disorder as a consequence of lung hypoplasia and diaphragm impairment, accompanying mild bronchostenosis and bronchial hyperreactivity after infections. 

A relevant aspect is the choice of surgical technique. The considerable issue that could diminish the risk of ingrowth of liver is proper distribution of sutures—not too close to the border of defect, and not too far from each other. An alternative could be a tile-like overlap arrangement of sutures, which could be a valuable amendment in CDH repair. This technique may prevent ingrowth of the liver to the thoracic cavity. Other technical modifications are continuous suture, horizontal mattress sutures, pledgeted interrupted sutures with specific, described by Kamran et al., sandwich-type buttressed repair [2]. They also reported omentum protrusion between simple, interrupted sutures before introducing modifications [2].

## 5. Conclusions

Thoracoscopy may be a useful and preferred technique in diagnosis and treatment. 

The thoracoscopy may be complemented with ultrasonography and multidetector computed tomography (MDCT) in the diagnostic process of questionable cases. The advantage of the endoscopic technique is the ability to confirm the diagnosis macroscopically or by taking tissue to histopathological examination and to treat the disease at the same time.

Minimally invasive techniques provide a precise, enlarged picture of anatomical structures so some difficult to identify details such as a small part of ingrowing liver could be identified and treated with improved precision. The concept of ‘ingrowing liver’ may be a possible explanation of a mechanism of the atypical diaphragmatic hernia recurrence in our case.

The aesthetic effect and small postoperative scarring also give the thoracoscopy an advantage over open surgery.

## Figures and Tables

**Table 1:** Timeline with relevant patient’s data.

Age	Patient’s Data
First day of life	The first thoracoscopy—primary closure of diaphragm defect
Between 0–4 years	Recurrent upper and lower respiratory tract infections
The first at four months, the second at two years old	Bronchoscopy
Almost four years old	Lobar pneumonia, X-ray
Four years and one month old	The ultrasonography of thoracic cavities during a control visit
Four years and two months old	The CT scan of thorax
Four years and six months old	The second thoracoscopy—removing the part of ‘ingrowing liver’ and suturing a diaphragmatic defect

## Data Availability

The datasets used and/or analyzed during the current study are available from the corresponding author on reasonable request.
